# Metabolism in the Niche: a Large-Scale Genome-Based Survey Reveals Inositol Utilization To Be Widespread among Soil, Commensal, and Pathogenic Bacteria

**DOI:** 10.1128/spectrum.02013-22

**Published:** 2022-08-04

**Authors:** Michael Weber, Thilo M. Fuchs

**Affiliations:** a Friedrich-Loeffler-Institut/Federal Research Institute for Animal Health, Institute of Molecular Pathogenesis, Jena, Germany; University of Nebraska—Lincoln

**Keywords:** *myo*-inositol, phytate, catabolic pathway, prevalence, ecological niche, bacterial metabolism, virulence, genomics, large-scale approach

## Abstract

Phytate is the main phosphorus storage molecule of plants and is therefore present in large amounts in the environment and in the diet of humans and animals. Its dephosphorylated form, the polyol *myo*-inositol (MI), can be used by bacteria as a sole carbon and energy source. The biochemistry and regulation of MI degradation were deciphered in Bacillus subtilis and Salmonella enterica, but a systematic survey of this catabolic pathway has been missing until now. For a comprehensive overview of the distribution of MI utilization, we analyzed 193,757 bacterial genomes, representing a total of 24,812 species, for the presence, organization, and taxonomic prevalence of inositol catabolic gene clusters (IolCatGCs). The genetic capacity for MI degradation was detected in 7,384 (29.8%) of all species for which genome sequences were available. IolCatGC-positive species were particularly found among *Actinobacteria* and *Proteobacteria* and to a much lesser extent in *Bacteroidetes*. IolCatGCs are very diverse in terms of gene number and functions, whereas the order of core genes is highly conserved on the phylum level. We predict that 111 animal pathogens, more than 200 commensals, and 430 plant pathogens or rhizosphere bacteria utilize MI, underscoring that IolCatGCs provide a growth benefit within distinct ecological niches.

**IMPORTANCE** This study reveals that the capacity to utilize inositol is unexpectedly widespread among soil, commensal, and pathogenic bacteria. We assume that this yet-neglected metabolism plays a pivotal role in the microbial turnover of phytate and inositols. The bioinformatic tool established here enables predicting to which extent and genetic variance a bacterial determinant is present in all genomes sequenced so far.

## INTRODUCTION

Inositol is the structural basis for many biomolecules, such as phosphatidylinositol, that belong to the components of eukaryotic cell membranes. Their mono-, di-, or triphosphorylated derivatives, the phosphoinositides, are membrane constituents that act as cytosolic solutes and contribute to cell signaling, cell motility, membrane trafficking, and phagocytosis (1). Inositol phosphates (InsPs) are synthesized in animals and plants as secondary messengers. InsPs have been reported to represent 2% to 60% of the total organic phosphorus in animal waste (2), pointing to their role in eutrophication of the environment. InsP_6_, phytate, is the main storage molecule of phosphorus and minerals in plants. InsPs play multiple roles in eukaryotic cell functions, including growth, lipid metabolism, and insulin sensitivity (3). The most prominent example is the second messenger, inositol-1,4,5-triphosphate (InsP_3_). Several intestinal pathogens, such as Salmonella spp., *Shigella* spp., Escherichia coli serotypes, and *Yersinia* spp., modulate the InsP metabolism of the host in different ways (4). InsPs exist in various forms of phosphorylation with one to six phosphates and isomeric forms such as d-*chiro*, *scyllo*, and *neo*, with *myo*-inositol hexakiphosphate (InsP_6_), or phytate, the most abundant form in terrestrial and aquatic environments (5), cells (1), and in the diet as well as in the gut (6).

Phytate is present in plant tissues such as bran and seeds of legumes and cereals, including oil seeds and nuts. The dephosphorylated form of phytate is *myo*-inositol (MI), a polyol that is a readily available carbon and energy source for microorganisms. So far, the capacity to catabolize MI has been experimentally demonstrated for a few bacteria only, including Rhizobium leguminosarum (7), Sinorhizobium meliloti (8), Lactobacillus casei (9), Klebsiella aerogenes (10), Corynebacterium glutamicum (11), Legionella pneumophila (12), Yersinia mollaretii (13), and Citrobacter koseri (14). The transporters, repressor, and enzymes involved in MI utilization have been investigated in detail for Bacillus subtilis (15, 16), whereas the enteropathogen Salmonella enterica was used to elucidate the complex regulatory network controlling the MI pathway (17–19).

As MI is the most abundant carbon source detectable in the soil (20), we assumed that MI utilization is a widespread metabolic capability in microbial communities. However, it is largely unknown to which extent aquatic, soil, plant-associated, and gut bacteria are able to degrade MI that was released from phytate or cellular components. Here, we predict that MI is a substrate that can be utilized by an unexpectedly large number of bacteria as a source of carbon and energy. We determine the number and prevalence of bacterial species and genomes carrying the genetic determinant required for the degradation of dephosphorylated inositols and discuss the critical role of bacteria in the global turnover of these compounds.

## RESULTS

### Search for *iol* genes and inositol catabolic gene clusters.

**FIG 1:**
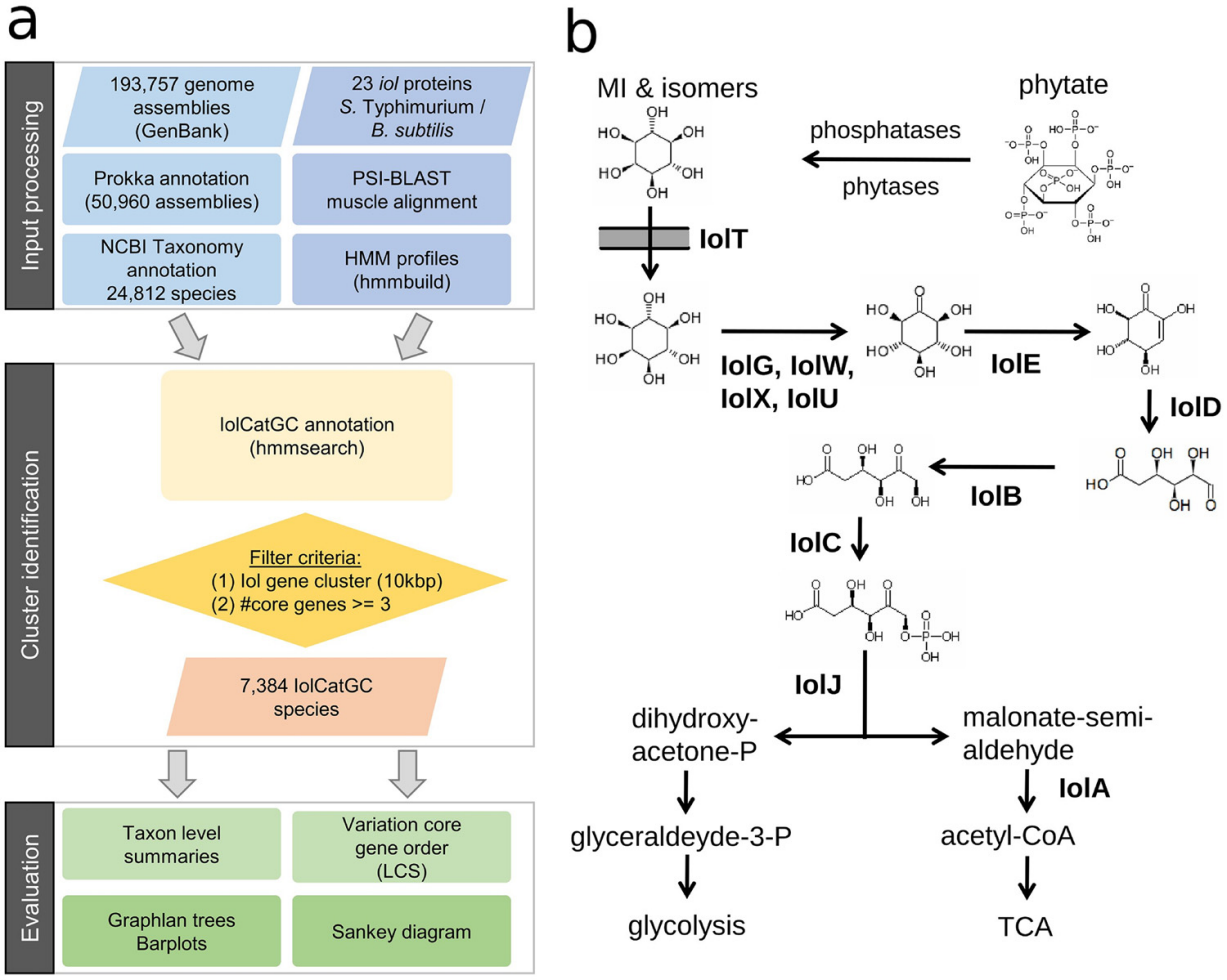
Searching for IolCatGCs. (a) The workflow depicts the bioinformatic strategy applied here to perform a comprehensive prediction of all bacteria able to degrade MI. Starting with the processing of 193,757 input genome assemblies, filtering resulted in the identification of an *iol* gene cluster termed IolCatGC whose prevalence was then evaluated at all taxonomic levels of bacteria. (b) A schematic pathway of inositol catabolism in Gram-positive and negative bacteria is shown. Enzymes catalyzing the degradation of MI to glyceraldehyde and acetyl-CoA are indicated. IolT, transporter; IolG, IolW, IolX, IolU, IolJ, dehydrogenases of inositol and its stereoisomers; IolE, dehydratase; IolD, 3D-(3,5/4)-trihydroxycyclohexane-1,2-dione hydrolase; IolB, isomerase; IolC, biphosphate aldolase; IolA, malonate-semialdehyde dehydrogenase; TCA, tricarboxylic acid cycle.

To obtain an overview of the distribution of inositol catabolic gene clusters (IolCatGCs), we performed a quality selection of bacterial genome sequences available in the GenBank database by applying RefSeq exclusion criteria, resulting in a total subset of 193,757 genomes that represent 24,812 species (Fig. 1a). In the case of 50,960 genome sequences, gene annotation files were not available in the database and were therefore generated using the annotation tool Prokka (21). Next, a basic set of 23 *iol* genes involved in the MI metabolism was taken from Salmonella enterica serovar Typhimurium and from B. subtilis, and its respective protein sequence was queried against the NCBI nonredundant (nr) database using PSI-BLAST. The resulting sequence hits were combined into hidden Markov models (HMMs) for each *iol* component. All 23 HMMs were searched against the total subset of genomes.

We defined a possibly functional IolCatGC to be present in a genome if at least three of the four core genes *iolB*, *iolC*, *iolD*, and *iolE*, which are essential for MI degradation (17) (Fig. 1b), were found in a distinct genetic determinant. Genes were assigned to an IolCatGC if they showed a maximum genetic distance of 10 kb. In 810 of 1,024 genomes with only 3 core genes, the 4th one was identified more than 10 kb apart from the IolCatGC elsewhere on the chromosome or within another contig, respectively. Possible reasons for a lack of the fourth gene are an incomplete genome sequence, assembly errors, or *iol* genes with frameshifts that were not considered to be functional. The genes *iolG1*, *iolG2*, *iolU*, and *iolW* encoding dehydrogenases of inositol isomers are expected to be present in genomes with a complete set of *iol* genes. Indeed, at least one of these dehydrogenase genes was identified in 6,502 IolCatGCs and outside the cluster in 881 species from our list. Genes encoding inositol transporters were not categorized as core genes due to their location apart from the IolCatGC in some bacteria (16) and not the genes encoding the malonate-semialdehyde dehydrogenase IolA and the biphosphate aldolase IolJ, which are also involved in the metabolism of valine or of fructose-1,6-biphosphate, respectively. The functions of genes associated with inositol catabolism are listed in Table S1 in the supplemental material. To not miss bacteria harboring an IolCatGC and to not erroneously state a species of not being capable of using MI, all genomes of strains belonging to the same species were analyzed for the presence and absence of *iol* genes. For each species with more than one genome available, we selected one IolCatGC that is representative in terms of cluster length and homology score (Table S2). We identified IolCatGCs within a total of 7,384 species corresponding to 29.8% of the 24,812 species’ genomes in the NCBI assembly database (Table S3). Among these are 3,651 Gram-negative and 3,733 Gram-positive species, corresponding to an almost equal distribution (14.7% versus 15%). Taken together, these findings highlight the relevance of MI degradation for many bacteria.

### Length, composition, and genetic organization of the IolCatGCs.

**FIG 2:**
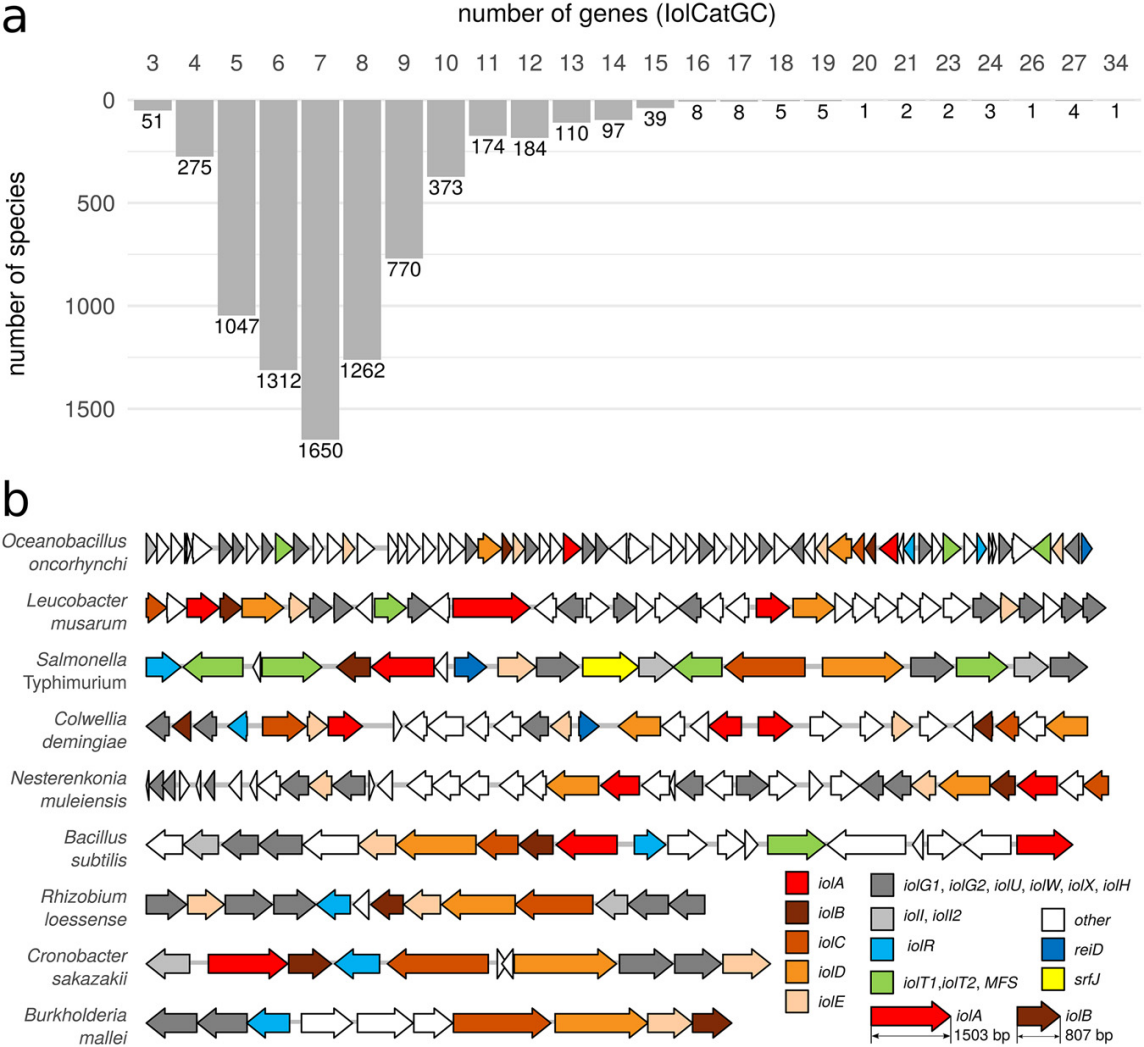
Complexity of the genetic IolCatGC organization. (a) The size of IolCatGCs in the genomes of bacterial species analyzed here is indicated by gene numbers. The species were considered that carry at least 3 and up to 34 genes within their IolCatGCs. The gene number was determined by counting genes encoding enzymes, regulators, transporters, and SrfJ, known or predicted to be involved in inositol utilization. Genes encoding transposases and hypothetical proteins or transporters without homology to putative Iol-degrading enzymes or characterized MI facilitators were not considered for defining the IolCatGC lengths. (b) Randomly selected IolCatGCs are depicted to illustrate their variability in length and composition. Regulators are stained blue; transporters, green; core genes, orange; and isomerases and dehydrogenases, gray. Numbers in brackets indicate the number of *iol* genes. White genes are genes of unknown function or not associated with inositol utilization.

We observed a wide range of complexity of IolCatGCs with respect to the number of operons and genes associated with inositol degradation. The vast majority, namely, 6,689 of the 7,384 IolCatGCs investigated, here, comprise 4 to 10 genes, while only 644 IolCatGCs comprise 11 to 34 genes (Fig. 2a). In addition to the core gene set, larger clusters contain genes coding for regulators, transporters, dehydrogenases, hypothetical proteins with related metabolic functions, and duplications thereof. These clusters appear to be organized as a divergon comprising at least two operons and promoters.

The genome of Oceanobacillus oncorhynchi carries a 76-kb fragment with 34 genes predicted to be involved in inositol degradation. Many functions, including those of the two regulators ReiD and IolR, which play a pivotal role in the regulation of MI degradation (19, 22, 23), are encoded twice, and 12 putative dehydrogenases providing the substrate for IolE were identified (Fig. 2b). Further examples randomly chosen are the IolCatGC from Leucobacter musarum, which was isolated from a nematode; Colwellia demingiae, a psychrophilic Antarctic species; and Nesterenkonia muleiensis, a novel actinobacterium isolated from Populus euphratica. Species of the latter genus are found in soil and are characterized by a high metabolic versatility. A more compact IolCatGC organization was found in the genomes of Rhizobium loessense, a root nodule bacterium; Cronobacter sakazakii, a foodborne pathogen; and Burkholderia mallei, a pathogen causing glanders. The IolCatGC of Salmonella enterica is more complex with respect to operon organization and gene numbers than most others. Its canonical *iol* gene cluster (17), together with that of B. subtilis (24), is also shown in Fig. 2b.

### Percentage of IolCatGC-positive genomes per species.

To exclude that a single genome erroneously represents a large number of IolCatGC-negative genomes, we analyzed the abundance of *iol* genes within all genomes available for each species. Out of the 7,384 species, 1,453 are represented by at least two genome sequences (Table S3a). In this group, we determined a high variability of a species pangenome with respect to the presence of *iol* genes. Regarding the 319 species for which at least 10 genome sequences met the quality criteria, 288 of them are represented by ≥0%, 246 by ≥50%, and 228 by ≥70% IolCatGC-positive genomes, respectively (Fig. S1; Table S3b). More than 1,000 genome sequences are available for 10 species (Table S3c). Among them are Listeria monocytogenes, Klebsiella pneumonia, Burkholderia pseudomallei, and Pseudomonas viridiflava, with at least 97% *iol* gene-positive sequences per species. Less than 2% of all 20,632 E. coli genome sequences comprise the IolCatGC.

### IolCatGCs encoding the activator ReiD and the putative ceramidase SrfJ.

**FIG 3:**
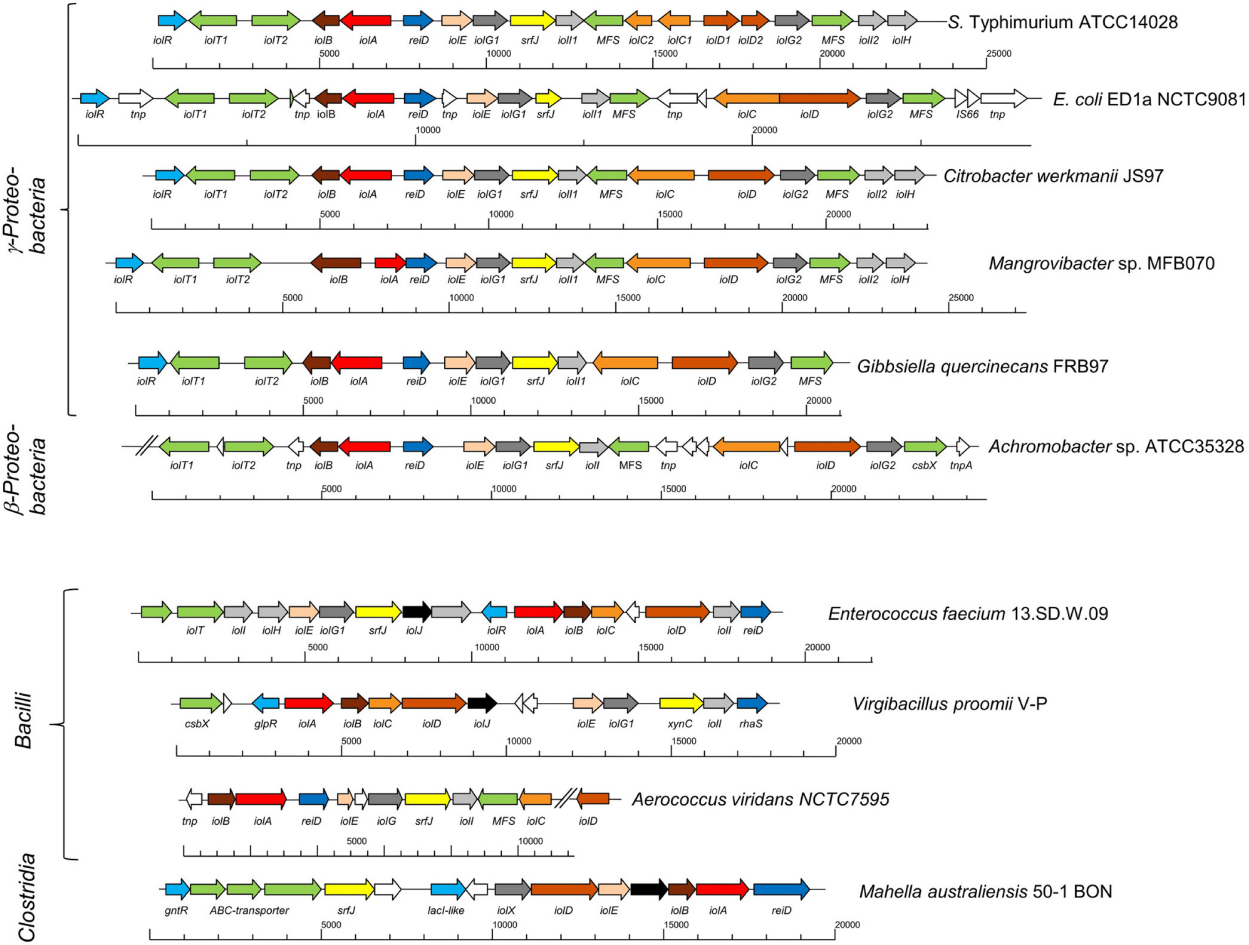
Complex IolCatGCs that harbor both the transcriptional activator gene *reiD* and the putative ceramidase gene *srfJ*. All IolCatGCs are shown that both carry the activator gene *reiD* and the putative ceramidase-encoding gene *srfJ*. The color code is the same as in Fig. 2. Genes encode transporters (green), core factors IolBCDE (orange), regulators (blue), the malonate-semialdehyde dehydrogenase IolA (red), the fructose-1,6-biphosphate aldolase IolJ (black), SrfJ (yellow), uncharacterized isomerases and dehydrogenases (gray), as well as transposases, insertion elements, genes not associated with MI utilization, and unknown genes (white). MFS, major facilitator superfamily; *csbX*, gene that encodes MFS efflux pump (69); *xynC*, *srfJ* homolog that encodes a glucuronoxylanase (70); *rhaS*, gene that codes for an activator of the l-rhamnose operon (71); *glpR*, gene coding for a repressor of sugar metabolism pathways (72); *gntR*, gene that encodes repressor of gluconate operon (73); *hyp*, hypothetical gene. Slashes indicate contig ends. Strain identification numbers or names are given. The classes the strains belong to are mentioned on the left.

Of particular interest are IolCatGC genes that are absent in the majority of IolCatGCs and encode accessory functions not essential for MI degradation. The most interesting ones are *reiD* and *srfJ*, which code for a regulator and a putative ceramidase, respectively. ReiD belongs to the AraC family of transcriptional regulators and was experimentally determined to activate the transcription of *iolE* and *iolG1*, the genes that encode the enzymes responsible for the initial two steps in MI degradation (19). Gene *srfJ* is known to be activated by the two-component system SsrAB, which controls the expression of SPI-2 genes (25, 26). Its product shows a remarkable similarity to human lysosomal glycosylceramidases (E value of 4 × 10^−62^; 30.8% amino acid identity) and might be involved in the release of inositols from sphingolipids. Homologs of ReiD were found in 217 species and those of SrfJ in 10 species among the list of 7,384 genomes with IolCatGCs, indicating that both factors are not widespread among bacteria. Remarkably, 10 IolCatGCs harbor both genes (Fig. 3). The clusters of Citrobacter werkmanii and *Mangrovibacter* spp. are identical to that of Salmonella and nearly colinear in E. coli, *Achromobacter* spp., and Gibbsiella quercinecans. In comparison with *S. Typhimurium*, the genomes of Gibbsiella quercinecans, *Achromobacter* sp., and Escherichia coli ED1a are lacking the two genes *iolI2* and *iolH*, and that of G. quercinecans a fourth transporter gene. The data suggest that additional regulatory, as well as enzymatic, determinants associated with MI degradation provide a benefit for some bacteria in their ecological niches.

### IolCatGC gene order.

**FIG 4:**
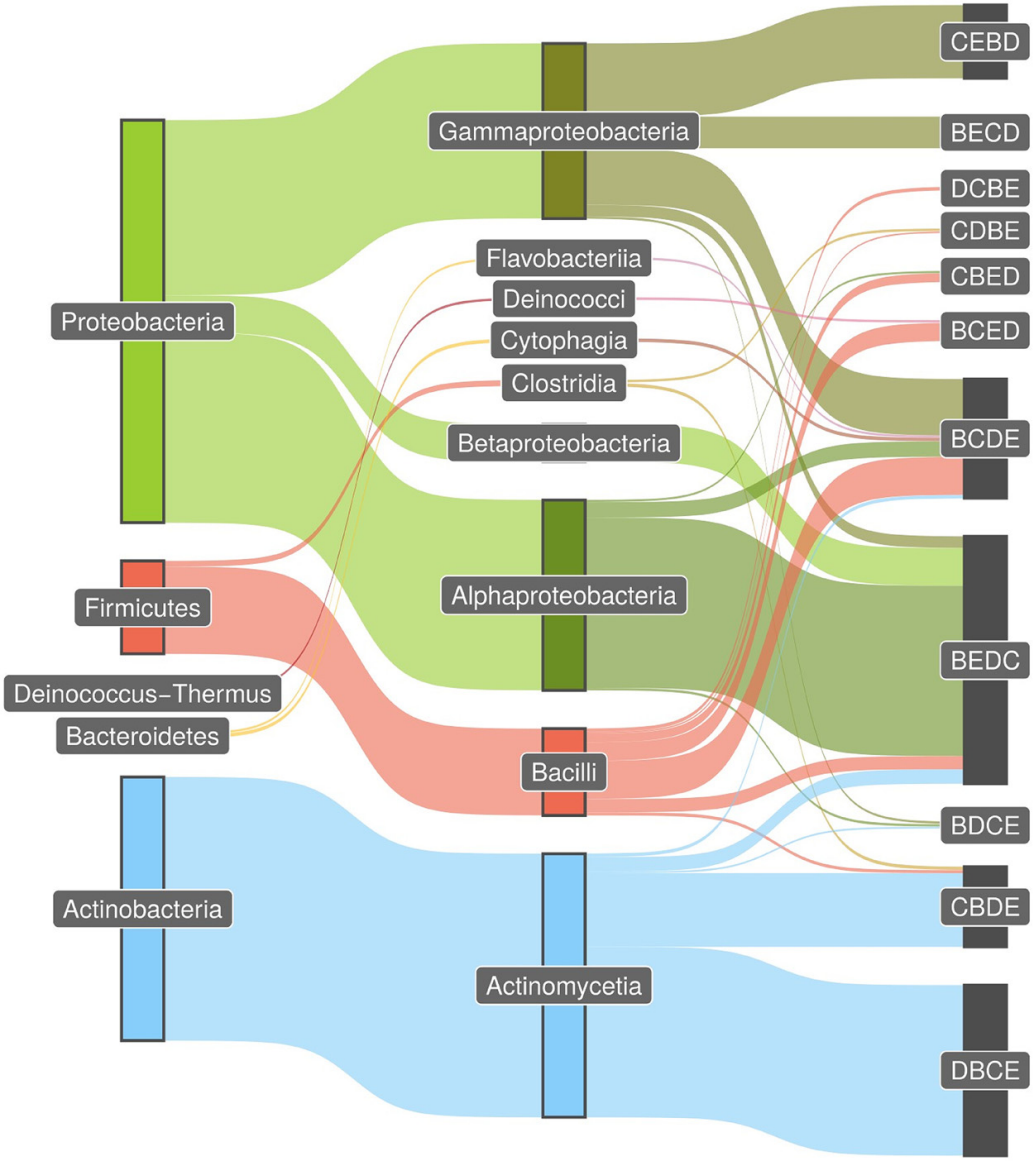
Patterns of *iol* gene order. The Sankey diagram was employed with the R package ggsankey and shows the most common order pattern of the core genes *iolB*, *iolC*, *iolD*, and *iolE* in the phylum and class levels as taxonomic nodes. The thickness of the connections corresponds with the relative proportion of species genomes carrying the same pattern. Genomes from five phyla were analyzed. In total, we found IolCatGCs in 6,026 species with unique pattern hits (81.6%) and in 548 (7.4%) species with multiple matches. Multiple matches are mainly due to the occurrence of multiple core genes in the cluster and could not be unequivocally assigned to one permutation type. A minimum of 10 species per type were required to draw a connection between taxonomic class and cluster type. In summary, we show connections for 80% (5,879) of the species in our results table.

Next, we investigated whether there are distinct patterns of IolCatGCs with respect to the genetic organization of the four core genes *iolB*, *iolC*, *iolD*, and *iolE*. Within the genomes of species belonging to the same genus, we predominantly observed collinearity of the genes involved in MI metabolism, pointing to a high degree of conservation on this taxonomic level (data not shown). Performing a systematic phyla evaluation (Fig. 4), we found that 65% of all IolCatGC-positive species belonging to *Actinobacteria* harbor the pattern *iolD*-*iolB*-*iolC*-*iolE* (DBCE) on their genomes, and 28% have the pattern CBDE (Table S4). The most common *iol* gene orders were BEDC in *Alphaproteobacteria* (42%) and CEBD (18%) and BCDE (14%) in *Gammaproteobacteria*. In *Firmicutes*, we mainly identified the gene orders BCDE, BCED, and BEDC, corresponding to 41%, 20%, and 14%, respectively, of all species in this phylum. The pattern BCDE was present in all genomes belonging to the classes *Cytophagia* and *Flavobacteria* (each 44%) of *Bacteroidetes* and was also common in *Chloroflexi* (data not shown). Thus, the *iol* gene orders BCDE and BEDC are interphylum patterns present in Gram-negative, as well as Gram-positive, bacteria.

To summarize, we observed a close relationship between taxonomy and pattern type, and we identified the pattern BEDC as the most abundant and widespread one. These data point to a highly conserved, ancient pathway that further evolved by the acquisition of regulatory, transporter, and nonessential enzymatic genes.

### Taxonomic prevalence of IolCatGC.

**FIG 5:**
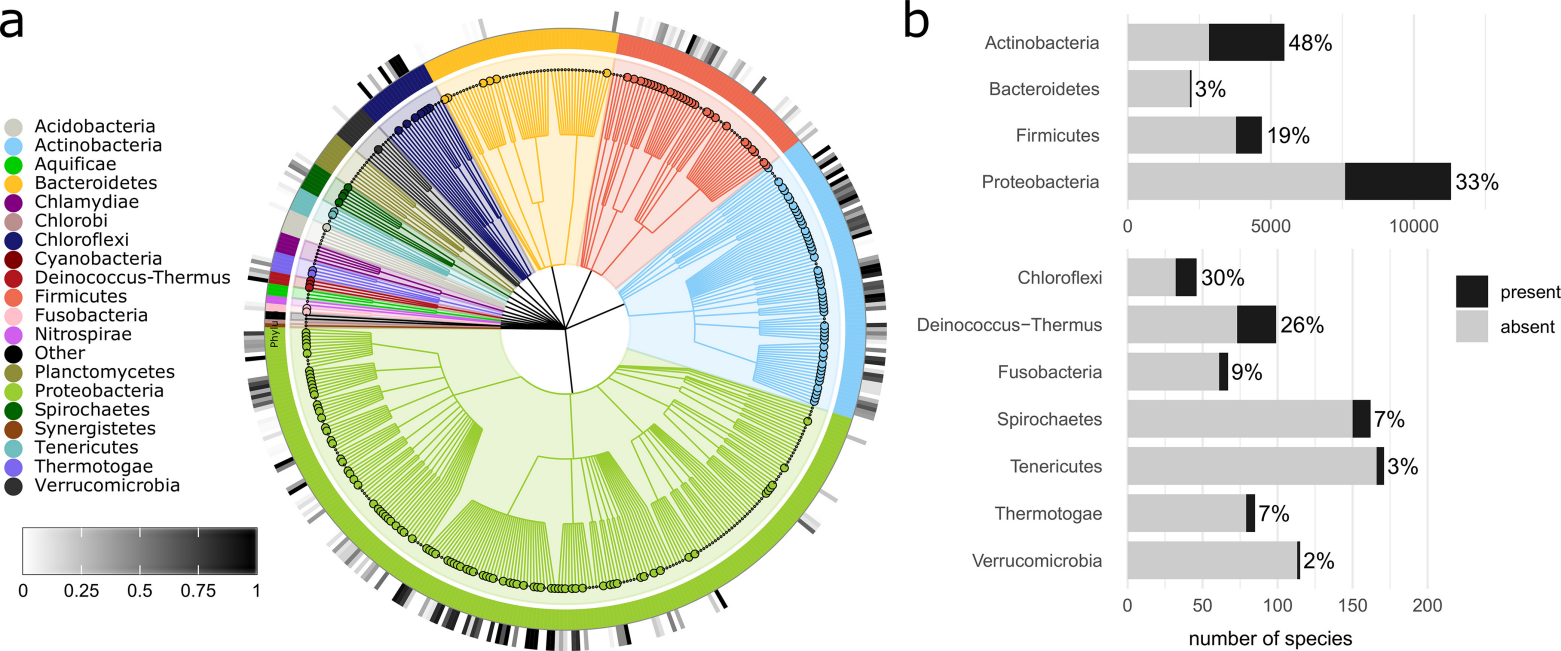
Prevalence of IolCatGCs among the bacterial kingdom. (a) Cladogram of analyzed bacterial species showing the prevalence of IolCatGCs at the family level. To filter out less relevant branches, only families with at least 10 species were considered. The taxonomic hierarchy includes phylum, class, order, and family (from inside to outside). Filled circles indicate families of which at least 10% of their species are IolCatGC positive. The outer circle heatmap indicates the percentage of species within a family that are IolCatGC positive. The figure was generated with GraPhlAn (version 1.1.4). (b) Bar plot indicating absolute and relative number of IolCatGC-positive species within selected bacterial phyla. The four largest phyla are shown in the upper part. The bar plot was generated with the help of R package ggplot2 (version 3.3.2).

To examine the distribution of *iol*-positive species within different taxonomic levels of bacteria, we defined a species as positive with respect to inositol catabolism if at least one genome per species carried an IolCatGC. We found IolCatGCs in species belonging to 12 out of 21 phyla (Fig. 5a; Table S5), with substantial proportions in *Actinobacteria* (48% out of 5,473 species), *Proteobacteria* (33% of 11,297), and *Firmicutes* (19% of 4,686). In the next largest phylum, *Bacteroidetes*, IolCatGCs were found in the genomes of only 3% out of 2,227 species (Fig. 5b). These data indicate an uneven distribution of IolCatGCs at the phylum level.

**FIG 6:**
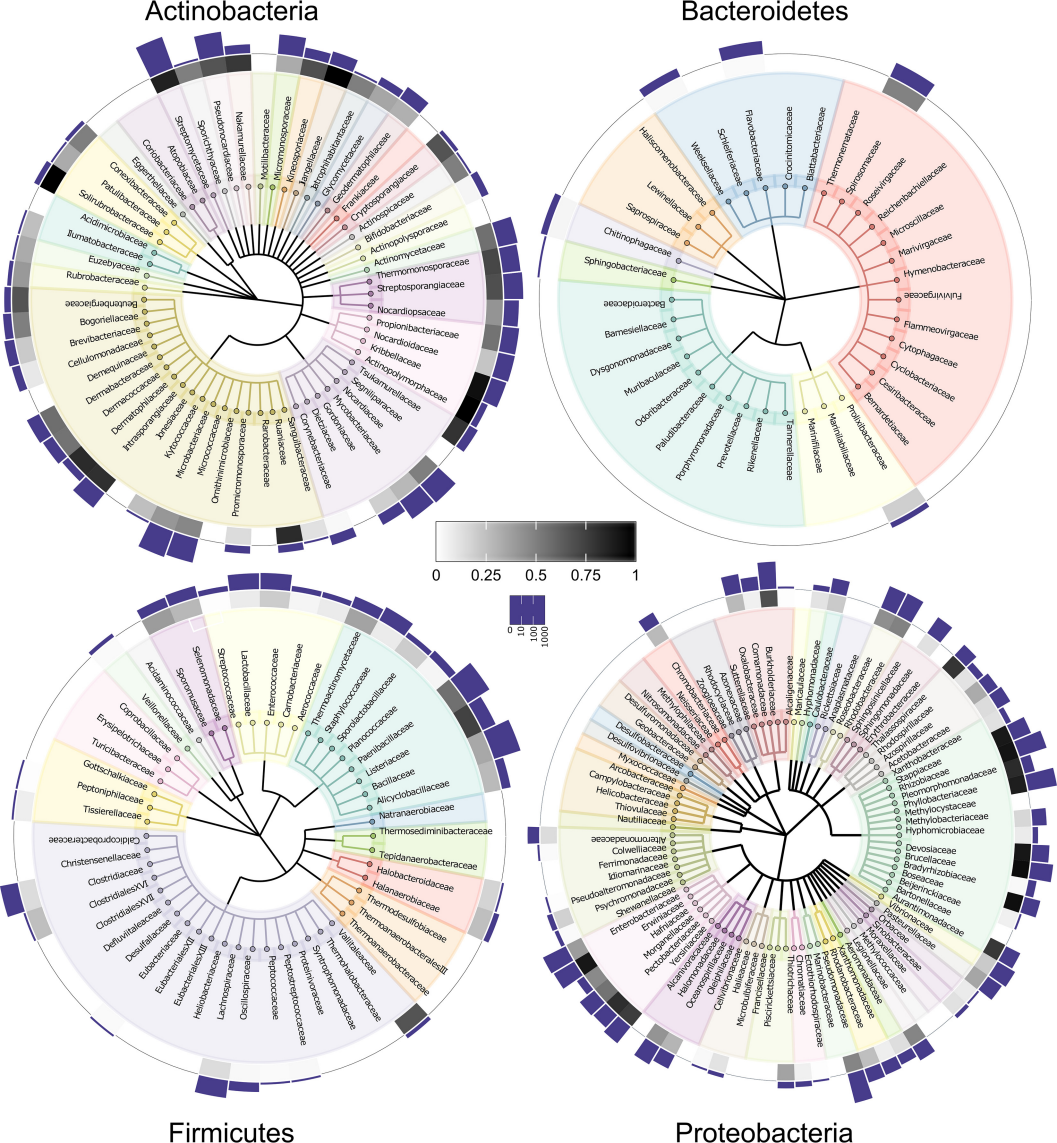
Families predicted to degrade MI. Families comprising at least 2 (*Actinobacteria*, *Bacteroidetes*, and *Firmicutes*) or at least 10 (*Proteobacteria*) species are indicated. The bars in the logarithmic scale show the total number, and the heatmap shows the percentage of species within a family that are IolCatGC positive. Scales are indicated.

On the family level, we identified IolCatGCs in 202 out of 473 bacterial families (Fig. 6). Analyzing the three largest families of the phylum *Actinobacteria*, we detected 87% IolCatGC positive out of 1,235 species of *Streptomycetaceae* dominated by the genus *Streptomyces*, 41% of 789 species belonging to *Microbacteriaceae*, and 28% of 395 species of *Mycobacteriaceae*. Fifty-six percent of the species belonging to *Alphaproteobacteria* and *Gammaproteobacteria* carry IolCatGCs. In particular, we identified *iol* genes in *Pseudomonadaceae* (48% of 1,221 species), *Enterobacteriaceae* (39% of 594 species), *Phyllobacteriaceae* (88% of 524 species), *Burkholderiaceae* (69% of 474 species), *Rhizobiaceae* (92% of 451 species), and *Vibrionaceae* (21% of 381 species). With respect to the phylum *Firmicutes*, we found IolCatGCs predominantly in *Bacillaceae* (34% of 1,078 species) and *Paenibacillaceae* (56% of 441 species) and, to lesser extent, in *Lactobacillaceae* (8% of 392 species), *Clostridiaceae* (14% of 352 species), *Lachnospiraceae* (14% of 324 species), and *Staphylococcaceae* (8% of 306 species). In *Bacteroidetes*, the capability of utilizing MI is less prevalent. IolCatGC-positive genomes were found in *Flavobacteriaceae* (2% of 810 species), *Weeksellaceae* (3% of 215 species), and *Spirosomaceae* (49% of 55 species), whereas no *iol* gene clusters were found in other 44 families, including the large families *Bacteroidaceae* and *Prevotellaceae*.

**FIG 7:**
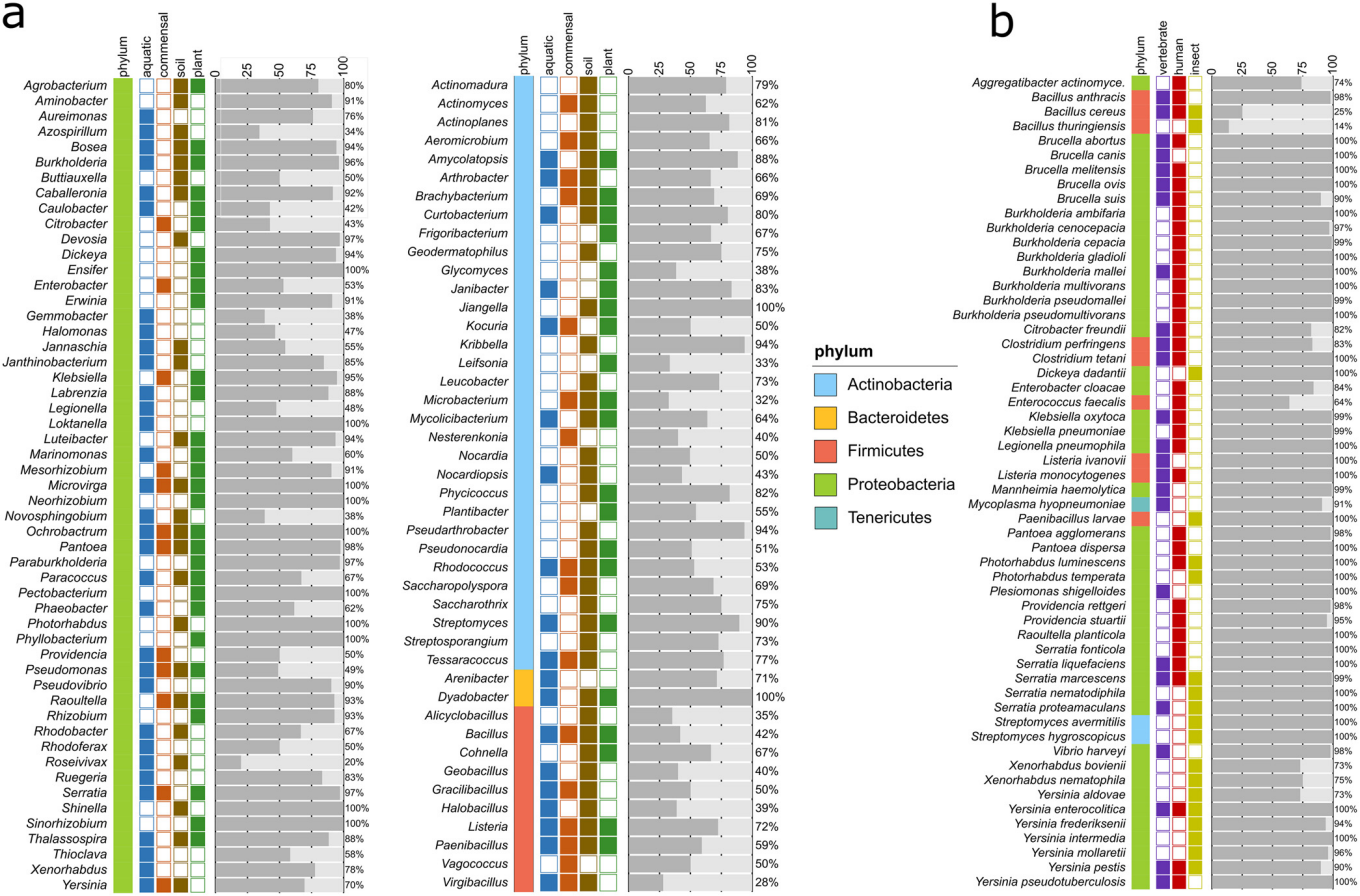
Distribution of the MI pathway at the genus level and in pathogenic bacteria. (a) Fraction of species within selected genera that carry an IolCatGC. Bacterial genera to which at least 10 species belong to, and of which ≥30% are IolCatGC positive, were selected. The number of species that carry (dark gray) or lack (light gray) the four core *iol* genes are shown. The site of sampling and/or typical ecological niche is indicated by colors. (b) Column plot showing the percentage of IolCatGC in all genomes of selected animal and human bacterial pathogens. Species for which at least 10 (insect pathogens, 5) genome sequences were available and of which at least 50% (with the exception of Bacillus cereus) were predicted as IolCatGC positive are shown.

The 7,384 species identified above belong to 776 genera (Table S6a). To analyze the most relevant ones with respect to inositol metabolism, we selected all genera that comprise at least 10 species of which ≥30% carry the IolCatGCs (Table S6b). These criteria are fulfilled by 97 genera (Fig. 7a). For 86 of them, at least 40% of their species are positive with respect to the *iol* gene cluster. Examples from this group are genera with at least 200 species, such as Pseudomonas, *Streptomyces*, *Bacillus*, *Mesorhizobium*, *Paenibacillus*, *Rhizobium*, *Burkholderia*, and *Rhodococcus*.

Taken together, the high abundance of IolCatGCs within these species-rich and other genera shown in Table S3 points out the relevance of MI degradation in particular for bacteria that are found in soil, water, decaying vegetation, the rhizosphere, or in association with plants. In these environments, phytate and MI are present in large amounts as accessible carbon and energy sources for bacteria.

### Plant pathogens and rhizosphere bacteria with *iol* genes.

From the list of IolCatGC-positive bacteria, we identified 82 species that are known as plant-pathogenic bacteria (27–29), most of them belonging to the genera *Agrobacterium*, *Burkholderia*, *Dickeya*, *Dyadobacter*, *Erwinia*, *Pantoea*, *Pectobacterium*, Pseudomonas, and *Ralstonia* (Table S7a). Among them are six of the top ten plant pathogens (30), namely A. tumefaciens, D. dadantii/solani, E. amylovora, P. carotovorum, P. syringae, and R. solanacearum (Fig. 7a). Another interesting group with respect to MI utilization are bacteria from the rhizosphere that promote plant growth by forming symbiotic root nodules. Six relevant genera from this group were therefore investigated for genomes with the capability of utilizing MI. Strikingly, IolCatGCs are highly prevalent in the genomes of 348 species (Table S7b) mainly belonging to *Caballeronia* (40 IolCatGC-positive genomes, corresponding to 93%), *Ensifer* (61 IolCatGC-positive genomes, corresponding to 100%), *Mesorhizobium* (459 IolCatGC-positive genomes, corresponding to 99%), *Paraburkholderia* (210 IolCatGC-positive genomes, corresponding to 100%), *Rhizobium* (829 IolCatGC-positive genomes, corresponding to 97%), and *Sinorhizobium* (305 IolCatGC-positive genomes, corresponding to 94%). To conclude, we hypothesize that these plant-associated bacteria gain a growth advantage by their inositol degradation capability in the phytate-rich plant environment.

### Commensals capable of degrading MI.

Given that phytate and inositol derivatives contribute to the diet and are ubiquitously present in the gut, we determined which and how many members of the microbiota in the gut carry IolCatGCs. For this purpose, we compiled a list of intestinal and rumen bacteria from humans, pigs, and cattle (Table S8). Our human gut microbiota list comprises a total of 807 nonredundant bacterial species (31, 32) and 465 swine species, including data from a DSMZ list (pig intestinal bacterial collection [PiBAC]) (33–37). A cattle reference list of 485 nonredundant rumen species was composed of the Hungate collection and genome sequencing projects (38–40). Remarkably, a high percentage of these species were found to carry IolCatGCs, namely, 16.6% of the human gut species, 10.3% of those from the swine gut, and 10.9% of all ruminal bacterial species identified so far. Examples of genera with *iol*-positive species among these commensals are *Bacillus*, *Blautia*, *Citrobacter*, *Clostridium*, *Corynebacterium*, Enterobacter, *Enterococcus*, Klebsiella, and *Paenibacillus* (Fig. 7a). IolCatGCs are absent, however, in Faecalibacterium prausnitzii in all but one *Bacteroides* spp., all but three *Bifidobacterium* spp., *Caprococcus* spp., and Akkermansia municiphila. From these data, we conclude that a substantial percentage of gut commensals, namely, at least 10%, are capable to degrade inositols and use them as an alternative substrate in the gut. Moreover, it might be speculated that the fraction of IolCatGC-positive commensals in the gut directly depends on the diet and the metabolic state of the host.

### Vertebrate pathogens harboring IolCatGC.

To identify bacterial pathogens equipped with *iol* genes, we compared Table S3 with a list of 636 species belonging to biosafety level 2 and 3 groups (Table S7c), resulting in a list of 87 species that are known as vertebrate pathogens, some of which are categorized as opportunistic, rare, or emerging pathogens (Table S7d). For an overview, we selected those 41 species for which ≥ 10 genomes were available, of which ≥ 50% carried IolCatGC (Fig. 7b; Table S7e and f). The genera Brucella, *Burkholderia*, *Clostridium*, Klebsiella, *Listeria*, *Pantoea*, *Providencia*, *Serratia*, and *Yersinia* are represented by two to eight species fulfilling these requirements. In addition, relevant pathogenic species such as Citrobacter freundii, Enterobacter cloacae, Enterococcus faecalis, Legionella pneumophila, Mannheimia haemolytica, S. enterica, and Vibrio harveyi were identified as encoding the capacity to utilize MI. Notably, most of those are enteropathogens. While 36% of all E. faecalis genomes do not carry *iol* genes, the genome sets of the other 41 pathogenic species shown in Fig. 7b with a proportion of ≥90% IolCatGC-positive sequences are much more coherent with respect to MI utilization. It is worth noting that the genes required for MI utilization are missing in the genera *Bartonella*, *Bordetella*, *Borrelia*, Campylobacter, Chlamydia, Mycobacterium, *Rickettsia*, and Streptococcus and in most Staphylococcus spp., including Staphylococcus aureus, all of which do not belong to *Enterobacteriaceae* and, with the exception of Campylobacter, do not proliferate in the gut.

To complement this survey on pathogens, we investigated the genomes of relevant bacterial insect pathogens (41–43) for the presence of IolCatGCs. We identified 20 entomopathogenic species without and 24 species with *iol* genes (Table S7g; Fig. 7b), among them, Bacillus thuringiensis, Photorhabdus luminescens, and Xenorhabdus nematophila, which play pivotal roles in pest control approaches. Paenibacillus larvae, the etiological agent of the American foul brood, colonizes the gut of honeybees and also carries an IolCatGC. Six insect-pathogenic *Yersinia* spp. (44) point to the fact that the interaction of species with invertebrates is, as of yet, underinvestigated.

Taken together, we identified a high number of pathogenic bacteria that are probably able to utilize MI and its derivatives. Given that the examples from above infect their hosts via the gut, lung, or bloodstream, we assume that the utilization of MI, which is present in food as well as in membrane compounds, provides a fitness advantage in different compartments of host organisms.

### MI utilization by archaea or fungi.

Fungi, in particular, yeasts, have been reported to utilize MI (45). For example, Cryptococcus neoformans is known to grow with inositol as a sole carbon and energy source (46). In contrast to the enzymes encoded by IolCatGC, the environmental and pathogenic yeast relies on an inositol oxygenase activity that is responsible for the conversion of inositol to d-glucuronic acid (47). However, to the best of our knowledge, there is no literature that describes a fungal MI degradation pathway with enzymatic functions similar to those in bacteria. It was reported that inositol induces the sporulation response of Beauveria bassiana and Metarhizium anisopliae (48). The genome of B. bassiana encodes proteins with similarity to IolR, IolG1, IolG2, and IolE (~25% sequence identity each); IolC (31%); IolD (45%); and IolB (32%) of Actinomyces ruminicola, but a reiterated search of these fungi-specific protein sequences did not result in increasing similarity rates. By analyzing 1,511 genomes of *Archaebacteria*, homologs of enzymes involved in MI degradation were identified in two genomes each of Halobacteriales archaeon and Desulfurococcaceae archaeon and in Thermocladium modestius (Table S8). Some of these sequences were derived from metagenomics, and experimental evidence for a functionality of the respective IolCatGC was not found.

We therefore conclude that, so far, the capability of degrading MI via IolCatGCs is restricted to members of the kingdom *Eubacteria*.

## DISCUSSION

The extensive genome sequences survey performed here required the public availability of hundreds of thousands of genome sequences, a well-adapted bioinformatics annotation pipeline, and the necessary parallel computing power. To our knowledge, this is one of the first comprehensive approaches that deciphers the prevalence and distribution of a single metabolic pathway across 24,812 bacterial species. A study addressing the highly complex production of the vitamin B_12_ family of cofactors investigated 11,000 bacterial species by comparative genomics (49). The annotation of IolCatGCs was based on stringent criteria to ensure high reliability of the results, including the definition of cluster core genes, a stringent cutoff for the HMM similarity score, and filtering for proximal localization of the annotated cluster components. The bioinformatics pipeline established here can be used to predict the prevalence of a selected bacterial pathway, genetic island, virulence factor, or any other genetic determinant in all taxonomic levels of bacteria. Moreover, the pipeline applied here and its data output can seamlessly be integrated into metagenomic studies.

Inositol phosphates accumulate in terrestrial environments where they constitute the major class of organic phosphorus compounds and are also present to great extent in aquatic environments (5). Thus, our finding that a huge number of bacterial genomes attributed to genera mainly found in the environment are potentially able to utilize MI is consistent with the presence of phytate and inositols in the environment (Fig. 6; see Table S6 in the supplemental material). Examples are Pseudomonas, *Streptomyces*, *Bacillus*, *Paenibacillus*, *Clostridium*, and *Halomonas* from saline environments and genera belonging to the ubiquitous *Actinobacteria* (*Amycolatopsis*, *Actinomadura*, *Actinomyces*, *Curtobacterium*, *Gordonia*, Mycobacterium, and *Rathayibacter*). Of particular interest is the microbiota of the rhizosphere for two reasons, namely, the availability of MI to promote bacterial growth and the dephosphorylation of phytate as organic phosphorus source (50). As expected, a majority of genomes belonging to main plant-associated bacterial genera carry the information to utilize MI. A benefit of inositol degradation was demonstrated for Rhizobium leguminosarum (7). This study supports the assumption that MI catabolism plays an important role for R. leguminosarum symbiosis with plants.

InsPs and phytates are widespread in organisms and in the diet, respectively, and MI is present in substantial amounts in the gut of humans and animals (51, 52). Indeed, we predicted more than 10% of commensal bacteria to be capable of utilizing MI for proliferation, indicating that IolCatGC is critical for some microbes to occupy microenvironments in the gut or to circumvent a depletion of other nutrients. In line with the substantial amount of IolCatGC-positive species frequently found in the gut, a metatranscriptome approach provided evidence that the capability of utilizing MI contributes to the bacterial fitness in a model of human gut microbial succession (53). When the metabolism of gut microbiota from mice was investigated by a metagenomic and metatranscriptomic approach, many *iol* genes were found to be differentially regulated in the presence of three antibiotics (54). These data corroborate *iol* gene activation in commensals and the functional role of the MI metabolism in the gut. Moreover, different amounts of mineral phosphorus and microbial phytases fed to chicken shaped the composition of their microbiota (55), and vegetarians’ microbiota revealed degradation of up to 100% phytate to MI-phosphate products lower than InsP_3_ (56). Therefore, it might be expected that nutrition affects the microbiological profile of gut microbiota in a IolCatGC-related manner. The two commensal species, Mitsuokella jalaludinii and Mitsuokella multacida, which are present in the porcine gastrointestinal tract as well as in the rumen of cattle (Tables S2 and S7), have been identified as antagonists of *S.* Typhimurium (57). As *Mitsuokella* spp. are able to degrade MI, it is tempting to speculate that the mechanism underlying this antagonism is based on a metabolic competition, which includes MI utilization.

Several pathogenic bacteria, mainly enteropathogens, are able to utilize MI and might thus gain an adaptive growth advantage during proliferation in gut niches in which other nutrients are not available or provide less energy than the polyol. Indeed, a transposon-directed insertion site sequencing (TraDIS)-based approach that systematically tested an *S.* Typhimurium transposon mutant library in chicken, pigs, and calves pointed to a strong attenuation of several *iol* gene mutants in enteritis models of these animals (58). Experimental data demonstrate that Legionella pneumophila utilizes MI to promote its infection of amoebae and macrophages (12).

### Conclusion.

Our comprehensive search for *iol* gene clusters exploited nearly 200,000 bacterial genomes and revealed that this metabolic capacity is more widely distributed among the bacterial kingdom than thought so far. Analysis of all bacterial taxonomic levels revealed an uneven distribution of the MI degradation pathway. Many soil and rhizosphere bacteria carry IolCatGCs in their genomes and benefit from this pathway due to the high concentration of phytate and inositol isomers in the environment. Remarkably, 10% to 16% of the human and animal microbiota members were identified as being capable of degrading MI, pointing to a metabolic niche that provides a growth advantage for gut bacteria. The presence of conserved *iol* gene clusters in one-quarter of all bacterial species sequenced so far strongly suggests that the MI degradation pathway plays a yet underestimated role in the metabolism and ecology of bacteria.

## MATERIALS AND METHODS

### Selection of genome assemblies.

Bacterial genome assemblies were downloaded from GenBank (accessed August 2020) using the public NCBI FTP server (ftp://ftp.ncbi.nlm.nih.gov/genomes/), including the nucleotide sequence fasta (fna), the annotated feature file (gff), and the translated protein sequences (faa) for each accession number. Assemblies were discarded if they matched a RefSeq exclusion criterion (https://www.ncbi.nlm.nih.gov/assembly/help/anomnotrefseq/). Genome assemblies with missing gene annotation files (50,960) were further analyzed for protein-coding sequences using Prokka (version 1.14.5) with default parameters (21).

### Genome data analysis.

The genome sequence of *S.* Typhimurium strain 14028 (GenBank accession no. NC_003197) was used to select the *iol* genes (*iolA*, *iolB*, *iolC*, *iolD*, *iolE*, *iolG1*, *iolG2*, *iolH*, *iolI*, *iolI2*, *srfJ*, *reiD*, *iolR*, *iolT1*, and *iolT2*) as an input (17). The genes *iolX*, *iolW*, *iolU*, *iolJ*, and *iolS* were taken from B. subtilis (GenBank accession no. NC_000964) (59, 60). For each gene associated with MI utilization (see Table S1 in the supplemental material), the following steps were applied. Translated protein sequences were queried against the NCBI nonredundant (nr) database using PSI-BLAST (61) with a total of 5 iterations; a cutoff of 70% percentage identity and 70% sequence coverage was chosen to select for highly similar sequences. The resulting list of orthologous protein sequences was aligned with the tool MUSCLE (62, 63), and the resulting multiple alignment was loaded into hmmbuild of package hmmer (version 3.3.2) to generate an *iol* gene-specific hidden Markov model (HMM) (64). In total, we obtained 23 HMMs for the gene cluster identification (Table S9).

### IolCatGC screening.

To implement a large-scale high-throughput search for IolCatGCs, we developed a custom R annotation pipeline which is based on a series of processing steps. For each genome, this included an import of the genome assembly file (gff) and translated protein sequences (faa) into R using packages rtracklayer and Biostrings (65). Next, hmmsearch applied the precomputed HMMs on the imported protein sequences with default parameters. Gram-positive strains were additionally queried with models *iolX*, *iolW*, *iolU*, *iolJ*, and *iolS*. All resulting hits were filtered by a stringent E value cutoff of 10^−10^. Next, all *iol* gene hits were assigned to gene clusters, which were identified by a minimum distance of 10 kbp between two consecutive hit genes on the same contig sequence. One representative cluster with the largest number of *iol* core genes (*iolC*, *iolB*, *IolD*, and *iolE*) and the largest number of total unique *iol* genes was selected for the assembly. This annotation procedure was applied to all input genome assemblies by using multicore functions of R package parallel.

### IolCatGC selection at the species level.

In order to select the most representative gene cluster of a species, we implemented a selection method which ranks the identified IolCatGC according to the following criteria: the most frequent IolCatGC size across all genomes per species, average HMM score, and a minimum number of three *iol* core genes. The top-ranked genome is considered the candidate IolCatGC representative genome for each species. The entire summary table, including information about all species, candidate IolCatGC size, and frequency of occurrence, is available as Table S2. Gene maps were generated with R package gggenes to display the composition and structure of selected clusters (66).

### Taxonomic analysis.

We used the taxonomic data supplied by the NCBI taxonomy database (http://www.ncbi.nlm.nih.gov/taxonomy). The two central database files, nodes.dmp and names.dmp, were downloaded (on 25 February 2022) and processed by the R package taxonomizr (version 0.5.3; https://github.com/sherrillmix/taxonomizr). These files provide hierarchical relationships between the taxonomic identity of species and strains and the respective taxonomic levels, including genus, family, order, class, and phylum. Mapping functions of the package provided the assignment of genome species IDs to their respective taxonomy. Only genomes with taxonomic assignments on all five levels were retained. Candidate species were not considered. All phylogenetic figures were generated with GraPhlAn (version 1.1.4) (67), requiring the generation of tree and annotation files as described (https://github.com/biobakery/graphlan).

### Cluster analysis.

The cluster analysis was done by comparing the core gene order in all IolCatGCs. To implement this comparison, we generated all possible permutations of the four core genes (*iolB*, *iolC*, *iolD*, and *iolE* [BCDE]) and removed reverse duplicates. As the gene sequence can be interrupted by other noncore genes, we used the LCS algorithm from R package qualV in forward and reverse directions to screen for clusters which are structured in the same order (68).

### Data availability.

All data are available in the tables in the supplemental material.

## References

[1] Michell RH. 2008. Inositol derivatives: evolution and functions. Nat Rev Mol Cell Biol 9:151–161. doi:10.1038/nrm2334. 18216771

[2] Peperzak P, Caldwell AG, Hunziker RR, Black CA. 1959. Phosphorus fractions in manures. Soil Science 87:293–302. doi:10.1097/00010694-195905000-00010.

[3] Huber K. 2016. Cellular *myo*-inositol metabolism, p 53–60. *In* Walk CL, Kühn I, Stein HH, Kidd MT, Rodehutscord M (ed), Phytate destruction: consequences for precision animal nutrition. Wageningen Academic Publishers, Wageningen, Netherlands.

[4] Heyer CM, Weiss E, Schmucker S, Rodehutscord M, Hoelzle LE, Mosenthin R, Stefanski V. 2015. The impact of phosphorus on the immune system and the intestinal microbiota with special focus on the pig. Nutr Res Rev 28:67–82. doi:10.1017/S0954422415000049. 26004147

[5] Turner BL, Paphazy MJ, Haygarth PM, McKelvie ID. 2002. Inositol phosphates in the environment. Philos Trans R Soc Lond B Biol Sci 357:449–469. doi:10.1098/rstb.2001.0837. 12028785 PMC1692967

[6] Staib L, Fuchs TM. 2014. From food to cell: nutrient exploitation strategies of enteropathogens. Microbiology (Reading) 160:1020–1039. doi:10.1099/mic.0.078105-0. 24705229

[7] Fry J, Wood M, Poole PS. 2001. Investigation of *myo*-inositol catabolism in *Rhizobium leguminosarum* bv. *viciae* and its effect on nodulation competitiveness. Mol Plant Microbe Interact 14:1016–1025. doi:10.1094/MPMI.2001.14.8.1016. 11497462

[8] Galbraith MP, Feng SF, Borneman J, Triplett EW, de Bruijn FJ, Rossbachl S. 1998. A functional *myo*-inositol catabolism pathway is essential for rhizopine utilization by *Sinorhizobium meliloti*. Microbiology 144:2915–2924. doi:10.1099/00221287-144-10-2915. 9802033

[9] Yebra MJ, Zuniga M, Beaufils S, Perez-Martinez G, Deutscher J, Monedero V. 2007. Identification of a gene cluster enabling *Lactobacillus casei* BL23 to utilize *myo*-inositol. Appl Environ Microbiol 73:3850–3858. doi:10.1128/AEM.00243-07. 17449687 PMC1932728

[10] Berman T, Magasanik B. 1966. The pathway of *myo*-inositol degradation in *Aerobacter aerogenes*. Ring scission. J Biol Chem 241:807–813. doi:10.1016/S0021-9258(18)96837-7. 5905123

[11] Krings E, Krumbach K, Bathe B, Kelle R, Wendisch VF, Sahm H, Eggeling L. 2006. Characterization of *myo*-inositol utilization by *Corynebacterium glutamicum*: the stimulon, identification of transporters, and influence on L-lysine formation. J Bacteriol 188:8054–8061. doi:10.1128/JB.00935-06. 16997948 PMC1698185

[12] Manske C, Schell U, Hilbi H. 2016. Metabolism of *myo*-inositol by *Legionella pneumophila* promotes infection of amoebae and macrophages. Appl Environ Microbiol 82:5000–5014. doi:10.1128/AEM.01018-16. 27287324 PMC4968532

[13] Wauters G, Janssens M, Steigerwalt AG, Brenner DJ. 1988. *Yersinia mollaretii* sp. nov. and *Yersinia bercovieri* sp. nov., formerly called *Yersinia enterocolitica* biogroups 3A and 3B. Int J Syst Evol Microbiol 38:424–429. doi:10.1099/00207713-38-4-424.

[14] Yuan C, Yang P, Wang J, Jiang L. 2019. *Myo*-inositol utilization by *Citrobacter koseri* promotes brain infection. Biochem Biophys Res Commun 517:427–432. doi:10.1016/j.bbrc.2019.07.112. 31376937

[15] Yoshida KI, Aoyama D, Ishio I, Shibayama T, Fujita Y. 1997. Organization and transcription of the *myo*-inositol operon, *iol*, of *Bacillus subtilis*. J Bacteriol 179:4591–4598. doi:10.1128/jb.179.14.4591-4598.1997. 9226270 PMC179296

[16] Yoshida K, Yamamoto Y, Omae K, Yamamoto M, Fujita Y. 2002. Identification of two *myo*-inositol transporter genes of *Bacillus subtilis*. J Bacteriol 184:983–991. doi:10.1128/jb.184.4.983-991.2002. 11807058 PMC134797

[17] Kröger C, Fuchs TM. 2009. Characterization of the *myo*-inositol utilization island of *Salmonella enterica* serovar Typhimurium. J Bacteriol 191:545–554. doi:10.1128/JB.01253-08. 19011032 PMC2620806

[18] Kröger C, Rothhardt JE, Brokatzky D, Felsl A, Kary SC, Heermann R, Fuchs TM. 2018. The small RNA RssR regulates *myo*-inositol degradation by *Salmonella enterica*. Sci Rep 8:17739. doi:10.1038/s41598-018-35784-8. 30531898 PMC6288124

[19] Rothhardt JE, Kröger C, Broadley SP, Fuchs TM. 2014. The orphan regulator ReiD of *Salmonella enterica* is essential for *myo*-inositol utilization. Mol Microbiol 94:700–712. doi:10.1111/mmi.12788. 25213016

[20] Fry J, Poole PS, Wood M. 1998. *myo*-inositol utilisation by *Rhizobium leguminosarum*. *In* Elmerich C, Kondorosi A, Newton WE (ed), Biological nitrogen fixation for the 21st century. Current plant science and biotechnology in agriculture, vol. 31. Springer Dordrecht, Dordrecht, Netherlands.

[21] Seemann T. 2014. Prokka: rapid prokaryotic genome annotation. Bioinformatics 30:2068–2069. doi:10.1093/bioinformatics/btu153. 24642063

[22] Hellinckx J, Heermann R, Felsl A, Fuchs TM. 2017. High binding activity of repressor IolR avoids costs of untimely induction of *myo*-inositol utilization by *Salmonella* Typhimurium. Sci Rep 7. doi:10.1038/srep44362. PMC5349611 28290506

[23] Kröger C, Srikumar S, Ellwart J, Fuchs TM. 2011. Bistability in *myo*-inositol utilization by *Salmonella enterica* serovar Typhimurium. J Bacteriol 193:1427–1435. doi:10.1128/JB.00043-10. 21239589 PMC3067638

[24] Yoshida K, Yamaguchi M, Morinaga T, Kinehara M, Ikeuchi M, Ashida H, Fujita Y. 2008. *myo*-Inositol catabolism in *Bacillus subtilis*. J Biol Chem 283:10415–10424. doi:10.1074/jbc.M708043200. 18310071

[25] Worley MJ, Ching KH, Heffron F. 2000. *Salmonella* SsrB activates a global regulon of horizontally acquired genes. Mol Microbiol 36:749–761. doi:10.1046/j.1365-2958.2000.01902.x. 10844662

[26] Cordero-Alba M, Bernal-Bayard J, Ramos-Morales F. 2012. SrfJ, a *Salmonella* type III secretion system effector regulated by PhoP, RcsB, and IolR. J Bacteriol 194:4226–4236. doi:10.1128/JB.00173-12. 22661691 PMC3416237

[27] Agrios GN. 2005. Plant pathology, 5th ed. Elsevier Academic Press, New York.

[28] Kim JS, Yoon SJ, Park YJ, Kim SY, Ryu CM. 2020. Crossing the kingdom border: human diseases caused by plant pathogens. Environ Microbiol 22:2485–2495. doi:10.1111/1462-2920.15028. 32307848

[29] Pscheidt JW. 2005. Pacific Northwest plant disease management handbook. Oregon State University, Corvallis, OR.

[30] Mansfield J, Genin S, Magori S, Citovsky V, Sriariyanum M, Ronald P, Dow M, Verdier V, Beer SV, Machado MA, Toth I, Salmond G, Foster GD. 2012. Top 10 plant pathogenic bacteria in molecular plant pathology. Mol Plant Pathol 13:614–629. doi:10.1111/j.1364-3703.2012.00804.x. 22672649 PMC6638704

[31] Zou Y, Xue W, Luo G, Deng Z, Qin P, Guo R, Sun H, Xia Y, Liang S, Dai Y, Wan D, Jiang R, Su L, Feng Q, Jie Z, Guo T, Xia Z, Liu C, Yu J, Lin Y, Tang S, Huo G, Xu X, Hou Y, Liu X, Wang J, Yang H, Kristiansen K, Li J, Jia H, Xiao L. 2019. 1,520 reference genomes from cultivated human gut bacteria enable functional microbiome analyses. Nat Biotechnol 37:179–185. doi:10.1038/s41587-018-0008-8. 30718868 PMC6784896

[32] Almeida A, Nayfach S, Boland M, Strozzi F, Beracochea M, Shi ZJ, Pollard KS, Sakharova E, Parks DH, Hugenholtz P, Segata N, Kyrpides NC, Finn RD. 2021. A unified catalog of 204,938 reference genomes from the human gut microbiome. Nat Biotechnol 39:105–114. doi:10.1038/s41587-020-0603-3. 32690973 PMC7801254

[33] Xiao L, Estelle J, Kiilerich P, Ramayo-Caldas Y, Xia Z, Feng Q, Liang S, Pedersen AO, Kjeldsen NJ, Liu C, Maguin E, Dore J, Pons N, Le Chatelier E, Prifti E, Li J, Jia H, Liu X, Xu X, Ehrlich SD, Madsen L, Kristiansen K, Rogel-Gaillard C, Wang J. 2016. A reference gene catalogue of the pig gut microbiome. Nat Microbiol 1:16161. doi:10.1038/nmicrobiol.2016.161. 27643971

[34] Wylensek D, Hitch TCA, Riedel T, Afrizal A, Kumar N, Wortmann E, Liu T, Devendran S, Lesker TR, Hernandez SB, Heine V, Buhl EM, M D'Agostino P, Cumbo F, Fischoder T, Wyschkon M, Looft T, Parreira VR, Abt B, Doden HL, Ly L, Alves JMP, Reichlin M, Flisikowski K, Suarez LN, Neumann AP, Suen G, de Wouters T, Rohn S, Lagkouvardos I, Allen-Vercoe E, Sproer C, Bunk B, Taverne-Thiele AJ, Giesbers M, Wells JM, Neuhaus K, Schnieke A, Cava F, Segata N, Elling L, Strowig T, Ridlon JM, Gulder TAM, Overmann J, Clavel T. 2020. A collection of bacterial isolates from the pig intestine reveals functional and taxonomic diversity. Nat Commun 11:6389. doi:10.1038/s41467-020-19929-w. 33319778 PMC7738495

[35] Fenske GJ, Ghimire S, Antony L, Christopher-Hennings J, Scaria J. 2020. Integration of culture-dependent and independent methods provides a more coherent picture of the pig gut microbiome. FEMS Microbiol Ecol 96:fiaa022. doi:10.1093/femsec/fiaa022. 32031212

[36] Jeong Y, Park J, Kim EB. 2020. Changes in gut microbial community of pig feces in response to different dietary animal protein media. J Microbiol Biotechnol 30:1321–1334. doi:10.4014/jmb.2003.03021. 32522966 PMC9728240

[37] Chen C, Zhou Y, Fu H, Xiong X, Fang S, Jiang H, Wu J, Yang H, Gao J, Huang L. 2021. Expanded catalog of microbial genes and metagenome-assembled genomes from the pig gut microbiome. Nat Commun 12:1106. doi:10.1038/s41467-021-21295-0. 33597514 PMC7889623

[38] Seshadri R, Leahy SC, Attwood GT, Teh KH, Lambie SC, Cookson AL, Eloe-Fadrosh EA, Pavlopoulos GA, Hadjithomas M, Varghese NJ, Paez-Espino D, Hungate project c, Perry R, Henderson G, Creevey CJ, Terrapon N, Lapebie P, Drula E, Lombard V, Rubin E, Kyrpides NC, Henrissat B, Woyke T, Ivanova NN, Kelly WJ, Hungate1000 Project Collaborators. 2018. Cultivation and sequencing of rumen microbiome members from the Hungate1000 Collection. Nat Biotechnol 36:359–367. doi:10.1038/nbt.4110. 29553575 PMC6118326

[39] Stewart RD, Auffret MD, Warr A, Wiser AH, Press MO, Langford KW, Liachko I, Snelling TJ, Dewhurst RJ, Walker AW, Roehe R, Watson M. 2018. Assembly of 913 microbial genomes from metagenomic sequencing of the cow rumen. Nat Commun 9:870. doi:10.1038/s41467-018-03317-6. 29491419 PMC5830445

[40] Stewart RD, Auffret MD, Warr A, Walker AW, Roehe R, Watson M. 2019. Compendium of 4,941 rumen metagenome-assembled genomes for rumen microbiome biology and enzyme discovery. Nat Biotechnol 37:953–961. doi:10.1038/s41587-019-0202-3. 31375809 PMC6785717

[41] Vallet-Gely I, Lemaitre B, Boccard F. 2008. Bacterial strategies to overcome insect defences. Nat Rev Microbiol 6:302–313. doi:10.1038/nrmicro1870. 18327270

[42] Ruiu L. 2015. Insect pathogenic bacteria in integrated pest management. Insects 6:352–367. doi:10.3390/insects6020352. 26463190 PMC4553484

[43] Galac MR, Lazzaro BP. 2011. Comparative pathology of bacteria in the genus *Providencia* to a natural host, *Drosophila melanogaster*. Microbes Infect 13:673–683. doi:10.1016/j.micinf.2011.02.005. 21354324 PMC3109104

[44] Fuchs TM, Bresolin G, Marcinowski L, Schachtner J, Scherer S. 2008. Insecticidal genes of *Yersinia* spp.: taxonomical distribution, contribution to toxicity towards *Manduca sexta* and *Galleria mellonella*, and evolution. BMC Microbiol 8:214. doi:10.1186/1471-2180-8-214. 19063735 PMC2613401

[45] Barnett JA. 1976. The utilization of sugars by yeasts. Adv Carbohydr Chem Biochem 32:125–234. doi:10.1016/s0065-2318(08)60337-6. 782183

[46] Healy ME, Dillavou CL, Taylor GE. 1977. Diagnostic medium containing inositol, urea, and caffeic acid for selective growth of *Cryptococcus neoformans*. J Clin Microbiol 6:387–391. doi:10.1128/jcm.6.4.387-391.1977. 334795 PMC274779

[47] Wang Y, Wear M, Kohli G, Vij R, Giamberardino C, Shah A, Toffaletti DL, Yu CA, Perfect JR, Casadevall A, Xue C. 2021. Inositol metabolism regulates capsule structure and virulence in the human pathogen *Cryptococcus neoformans*. mBio 12:e0279021. doi:10.1128/mBio.02790-21. 34724824 PMC8561382

[48] Campbell RK, Barnes GL, Cartwright BO, Eikenbary RD. 1983. Growth and sporulation of *Beauveria bassiana* and *Metarhizium anisopliae* in a basal medium containing various carbohydrate sources. J Invert Pathol 41:117–121. doi:10.1016/0022-2011(83)90242-2.

[49] Shelton AN, Seth EC, Mok KC, Han AW, Jackson SN, Haft DR, Taga ME. 2019. Uneven distribution of cobamide biosynthesis and dependence in bacteria predicted by comparative genomics. ISME J 13:789–804. doi:10.1038/s41396-018-0304-9. 30429574 PMC6461909

[50] Unno Y, Shinano T. 2013. Metagenomic analysis of the rhizosphere soil microbiome with respect to phytic acid utilization. Microbes Environ 28:120–127. doi:10.1264/jsme2.me12181. 23257911 PMC4070688

[51] Matsumoto M, Kibe R, Ooga T, Aiba Y, Kurihara S, Sawaki E, Koga Y, Benno Y. 2012. Impact of intestinal microbiota on intestinal luminal metabolome. Sci Rep 2:233. doi:10.1038/srep00233. 22724057 PMC3380406

[52] Sommerfeld V, Kunzel S, Schollenberger M, Kuhn I, Rodehutscord M. 2018. Influence of phytase or *myo*-inositol supplements on performance and phytate degradation products in the crop, ileum, and blood of broiler chickens. Poult Sci 97:920–929. doi:10.3382/ps/pex390. 29300969 PMC5850709

[53] Feng L, Raman AS, Hibberd MC, Cheng J, Griffin NW, Peng Y, Leyn SA, Rodionov DA, Osterman AL, Gordon JI. 2020. Identifying determinants of bacterial fitness in a model of human gut microbial succession. Proc Natl Acad Sci USA 117:2622–2633. doi:10.1073/pnas.1918951117. 31969452 PMC7007522

[54] Cabral DJ, Penumutchu S, Reinhart EM, Zhang C, Korry BJ, Wurster JI, Nilson R, Guang A, Sano WH, Rowan-Nash AD, Li H, Belenky P. 2019. Microbial metabolism modulates antibiotic susceptibility within the murine gut microbiome. Cell Metab 30:800–823.e7. doi:10.1016/j.cmet.2019.08.020. 31523007 PMC6948150

[55] Tilocca B, Witzig M, Rodehutscord M, Seifert J. 2016. Variations of phosphorous accessibility causing changes in microbiome functions in the gastrointestinal tract of chickens. PLoS One 11:e0164735. doi:10.1371/journal.pone.0164735. 27760159 PMC5070839

[56] Markiewicz LH, Honke J, Haros M, Świątecka D, Wróblewska B. 2013. Diet shapes the ability of human intestinal microbiota to degrade phytate–in vitro studies. J Appl Microbiol 115:247–259. doi:10.1111/jam.12204. 23551617

[57] Levine UY, Bearson SM, Stanton TB. 2012. *Mitsuokella jalaludinii* inhibits growth of *Salmonella enterica* serovar Typhimurium. Vet Microbiol 159:115–122. doi:10.1016/j.vetmic.2012.03.027. 22503601

[58] Chaudhuri RR, Morgan E, Peters SE, Pleasance SJ, Hudson DL, Davies HM, Wang J, van Diemen PM, Buckley AM, Bowen AJ, Pullinger GD, Turner DJ, Langridge GC, Turner AK, Parkhill J, Charles IG, Maskell DJ, Stevens MP. 2013. Comprehensive assignment of roles for *Salmonella* Typhimurium genes in intestinal colonization of food-producing animals. PLoS Genet 9:e1003456. doi:10.1371/journal.pgen.1003456. 23637626 PMC3630085

[59] Morinaga T, Ashida H, Yoshida KI. 2010. Identification of two *scyllo*-inositol dehydrogenases in *Bacillus subtilis*. Microbiology (Reading) 156:1538–1546. doi:10.1099/mic.0.037499-0. 20133360

[60] Kang DM, Tanaka K, Takenaka S, Ishikawa S, Yoshida KI. 2017. *Bacillus subtilis iolU* encodes an additional NADP(+)-dependent scyllo-inositol dehydrogenase. Biosci Biotechnol Biochem 81:1026–1032. doi:10.1080/09168451.2016.1268043. 28043209

[61] Altschul SF, Madden TL, Schaffer AA, Zhang J, Zhang Z, Miller W, Lipman DJ. 1997. Gapped BLAST and PSI-BLAST: a new generation of protein database search programs. Nucleic Acids Res 25:3389–3402. doi:10.1093/nar/25.17.3389. 9254694 PMC146917

[62] Edgar RC. 2004. MUSCLE: a multiple sequence alignment method with reduced time and space complexity. BMC Bioinformatics 5:113. doi:10.1186/1471-2105-5-113. 15318951 PMC517706

[63] Edgar RC. 2004. MUSCLE: multiple sequence alignment with high accuracy and high throughput. Nucleic Acids Res 32:1792–1797. doi:10.1093/nar/gkh340. 15034147 PMC390337

[64] Eddy SR. 2009. A new generation of homology search tools based on probabilistic inference. Genome Inform 23:205–211. doi:10.1142/9781848165632_0019. 20180275

[65] Pagès H, Aboyoun P, Gentleman R, DebRoy S. 2020. Biostrings: efficient manipulation of biological strings. R package version 2.58.0.

[66] Wilkins D. 2020. Gggenes: drawgene arrowmaps in 'ggplot2'. R package version 041.

[67] Asnicar F, Weingart G, Tickle TL, Huttenhower C, Segata N. 2015. Compact graphical representation of phylogenetic data and metadata with GraPhlAn. PeerJ 3:e1029. doi:10.7717/peerj.1029. 26157614 PMC4476132

[68] Jachner S, Boogaart G, Petzoldt T. 2007. Statistical methods for the qualitative assessment of dynamic models with time delay (R Package qualV). J Stat Soft 22:1–30. doi:10.18637/jss.v022.i08.

[69] Page WJ, Kwon E, Cornish AS, Tindale AE. 2003. The *csbX* gene of *Azotobacter vinelandii* encodes an MFS efflux pump required for catecholate siderophore export. FEMS Microbiol Lett 228:211–216. doi:10.1016/S0378-1097(03)00753-5. 14638426

[70] St John FJ, Rice JD, Preston JF. 2006. Characterization of XynC from *Bacillus subtilis* subsp. *subtilis* strain 168 and analysis of its role in depolymerization of glucuronoxylan. J Bacteriol 188:8617–8626. doi:10.1128/JB.01283-06. 17028274 PMC1698249

[71] Tobin JF, Schleif RF. 1987. Positive regulation of the *Escherichia coli* L-rhamnose operon is mediated by the products of tandemly repeated regulatory genes. J Mol Biol 196:789–799. doi:10.1016/0022-2836(87)90405-0. 3316663

[72] Schweizer HP, Po C. 1996. Regulation of glycerol metabolism in *Pseudomonas aeruginosa*: characterization of the *glpR* repressor gene. J Bacteriol 178:5215–5221. doi:10.1128/jb.178.17.5215-5221.1996. 8752340 PMC178319

[73] Hoskisson PA, Rigali S. 2009. Chapter 1: variation in form and function of the helix-turn-helix regulators of the GntR superfamily. Adv Appl Microbiol 69:1–22. doi:10.1016/S0065-2164(09)69001-8. 19729089

