# Supplementary material for: Metabolism in the Niche: a Large-Scale Genome-Based Survey Reveals Inositol Utilization To Be Widespread among Soil, Commensal, and Pathogenic Bacteria
Source: Microbiol Spectr. 2022 Aug 4;10(4):e02013-22. doi: 10.1128/spectrum.02013-22 (PMC9430895; doi:10.1128/spectrum.02013-22)
Supplement: Supplemental file 1 — Fig. S1, Table S1, and list of additional files. Download spectrum.02013-22-s0001.pdf, PDF file, 0.9 MB [file spectrum.02013-22-s0001.pdf]

Additional files

FIG. S1: IolCatGC-positive genomes per species. The dot plot indicates the relative prevalence of *iol* gene-positive genomes belonging to the same species (left). The total number of genomes available for one species is given in a logarithmic scale. For most species, one representative genome sequence only was found in the databases (relative prevalence = 1). The colors indicated the phylum to which a genome sequence set belongs to (right). The plot was generated with the help of R package ggplot2 (3.3.2).

Table S1: Functions encoded by *iol* genes

xlsx-files:

Table S2: Summary table with representative species

Table S3: IolCatGC-positive species

Table S4: Values Sankey diagram

Table S5: Bacterial phyla overview of IolCatGC-positive species

Table S6: IolCatGC-positive species in bacterial genera

Table S7: Pathogenic bacteria harbouring IolCatGC

Table S8: Gut commensals harbouring IolCatGC

Table S9: Archaea with Iol protein homologs

FIG. S1: IolCatGC-positive genomes per species.

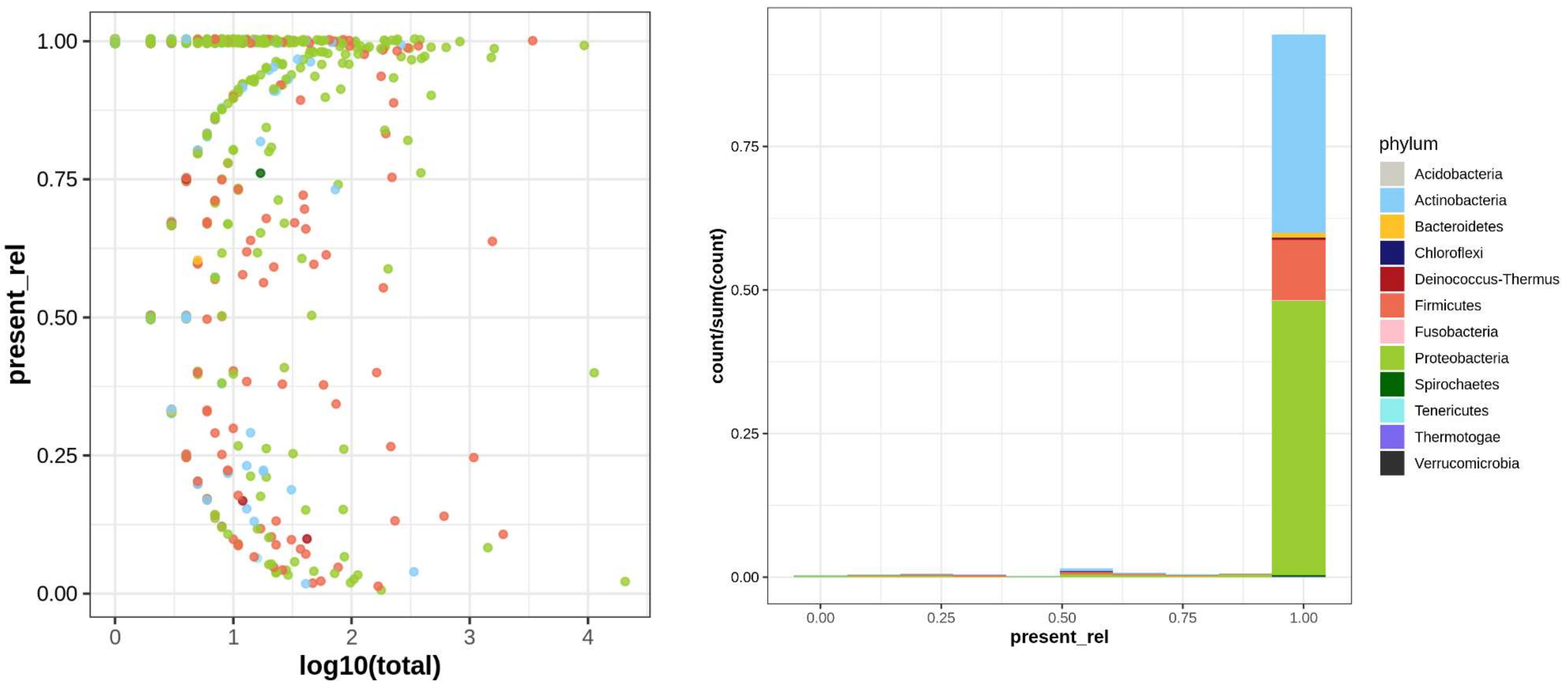

Table S1: Proteins and functions encoded by *iol* gene cluster

Gene	Protein	Function	Literature
iolA	Malonate-semialdehyde dehydrogenase	Conversion of malonic semialdehyde (MSA) to acetyl-CoA + CO_2	[1]
iolB	Isomerase	Isomerisation of 5-deoxy-D-glucuronic acid to 2-deoxy-5-keto-D-gluconic acid (DKG)	[1]
iolC	5-dehydro-2-deoxygluconokinase	Phosphorylation of DKG to DKG 6-phosphate	[1]
iolD	3D-(3,5/4)-trihydroxycyclohexane-1,2-dione hydrolase	Hydrolysis of 3D-(3,5/4)- Yoshida trihydroxycyclohexane-1,2-dione (THcHDO) to 5-deoxy-D-glucuronic acid	[1]
iolE	Dehydratase	Dehydratation of 2-keto-MI (2KMI) to THcHDO	[2]
iolG1	Inositol-dehydrogenase	Oxidation of of MI to 2KMI	[1]
iolG2	D-chiro-inositol-2-dehydrogenase	Oxidation of D-chiro-inositol to 1-keto-D-chiro-inositol	-
iolH	Isomerase/dehydratase	Unknown	[1]
iolI1	Isomerase	Interconverts 2KMI and 1-keto-D-chiro-inositol	[1]
iolJ	Fructose-1,6-biphosphate aldolase	Cleavage of DKGP into dihydroxyaceton-phosphate and MSA	[1]
iolR	Transcriptional regulator	Repressor of *iol* genes	[3, 4]
iolS	Pyridoxal reductase	Unknown	[1]
iolT1	Permease	Uptake of MI	[5, 6]
iolT2	Permease	Uptake of MI	[5]
iolU	Scyllo-inositol dehydrogenase	Dehydrogenation of scyllo-inositol to 2KMI	[7]
iolW	Scyllo-inositol dehydrogenase	Dehydrogenation of scyllo-inositol to 2KMI	[8]
iolX	Scyllo-inositol dehydrogenase	Dehydrogenation of scyllo-inositol to 2KMI	[8]
reiD	Transcriptional regulator	Activation of P_{iolEG1}	[9]
srfJ	putative lysosomale glykosylceramidase	Unknown, induced by SsrB	[10]

References:

1. Yoshida K, Yamaguchi M, Morinaga T, Kinehara M, Ikeuchi M, Ashida H *et al. myo*-Inositol catabolism in *Bacillus subtilis*. J Biol Chem 2008; 283:10415-10424.
2. Yoshida K, Yamaguchi M, Ikeda H, Omae K, Tsurusaki K, Fujita Y. The fifth gene of the iol operon of *Bacillus subtilis*, *iolE*, encodes 2-keto-*myo*-inositol dehydratase. Microbiology 2004; 150:571-580.
3. Yoshida KI, Shibayama T, Aoyama D, Fujita Y. Interaction of a repressor and its binding sites for regulation of the *Bacillus subtilis iol* divergon. J Mol Biol 1999; 285:917-929.
4. Kröger C, Fuchs TM. Characterization of the *myo*-inositol utilization island of *Salmonella enterica* serovar Typhimurium. J Bacteriol 2009; 191:545-554.
5. Yoshida K, Yamamoto Y, Omae K, Yamamoto M, Fujita Y. Identification of two *myo*-inositol transporter genes of *Bacillus subtilis*. J Bacteriol 2002; 184:983-991.
6. Kröger C, Stolz J, Fuchs TM. *myo*-Inositol transport by *Salmonella enterica* serovar Typhimurium. Microbiology 2010; 156:128-138.
7. Kang DM, Tanaka K, Takenaka S, Ishikawa S, Yoshida KI. *Bacillus subtilis iolU* encodes an additional NADP(+)-dependent scyllo-inositol dehydrogenase. Biosci Biotechnol Biochem 2017; 81:1026-1032.
8. Morinaga T, Ashida H, Yoshida KI. Identification of two *scyllo*-inositol dehydrogenases in *Bacillus subtilis*. Microbiology 2010; 156:1538-1546.
9. Rothhardt JE, Kröger C, Broadley SP, Fuchs TM. The orphan regulator ReiD of *Salmonella enterica* is essential for *myo*-inositol utilization. Mol Microbiol 2014; 94:700-712.
10. Worley MJ, Ching KH, Heffron F. *Salmonella* SsrB activates a global regulon of horizontally acquired genes. Mol Microbiol 2000; 36:749-761.
